# Nutritional and Microbiota-Based Approaches in Amyotrophic Lateral Sclerosis: From Prevention to Treatment

**DOI:** 10.3390/nu17010102

**Published:** 2024-12-30

**Authors:** Francesca Cuffaro, Ingrid Lamminpää, Elena Niccolai, Amedeo Amedei

**Affiliations:** 1 Division of Interdisciplinary Internal Medicine, Careggi University Hospital of Florence, 50134 Florence, Italy; cuffarof@aou-careggi.toscana.it; 2 Department of Experimental and Clinical Medicine, University of Florence, 50134 Firenze, Italy; ingrid.lamminpaa@unifi.it (I.L.); amedeo.amedei@unifi.it (A.A.); 3 Laboratorio Congiunto MIA-LAB (Microbiome-Immunity Axis Research for a Circular Health), University of Florence, 50134 Firenze, Italy; 4 Network of Immunity in Infection, Malignancy and Autoimmunity (NIIMA), Universal Scientific Education and Research Network (USERN), 50139 Florence, Italy

**Keywords:** metabolism, lipid, microbiome, neuroinflammation, immunity, nutrition

## Abstract

Metabolic alterations, including hypermetabolism, lipid imbalances, and glucose dysregulation, are pivotal contributors to the onset and progression of Amyotrophic Lateral Sclerosis (ALS). These changes exacerbate systemic energy deficits, heighten oxidative stress, and fuel neuroinflammation. Simultaneously, gastrointestinal dysfunction and gut microbiota (GM) dysbiosis intensify disease pathology by driving immune dysregulation, compromising the intestinal barrier, and altering gut–brain axis (GBA) signaling, and lastly advancing neurodegeneration. Therapeutic and preventive strategies focused on nutrition offer promising opportunities to address these interconnected pathophysiological mechanisms. Diets enriched with antioxidants, omega-3 fatty acids, and anti-inflammatory compounds—such as the Mediterranean diet—have shown potential in reducing oxidative stress and systemic inflammation. Additionally, microbiota-targeted approaches, including probiotics, prebiotics, postbiotics, and fecal microbiota transplantation, are emerging as innovative tools to restore microbial balance, strengthen gut integrity, and optimize GBA function. This review highlights the critical need for personalized strategies integrating immunonutrition and microbiota modulation to slow ALS progression, improve quality of life, and develop preventive measures for neurodegenerative and neuroinflammatory diseases. Future research should prioritize comprehensive dietary and microbiota-based interventions to uncover their therapeutic potential and establish evidence-based guidelines for managing ALS and related disorders.

## 1. Introduction

Amyotrophic lateral sclerosis (ALS) is a fatal neurodegenerative disease characterized by the progressive degeneration of upper and lower motor neurons leading to muscle weakness, atrophy, and paralysis [1]. As the disease progresses, patients experience a loss of voluntary muscle control, lastly affecting respiratory muscles and leading to respiratory failure, which is the primary cause of death. Despite extensive research, ALS remains poorly understood, with survival time typically ranging from 20 to 48 months following symptom onset, and only 5–10% of patients surviving more than 10 years [2]. The disease is highly heterogeneous, both in terms of phenotipic presentation and rate of progression [2]. This variability in ALS makes its diagnosis challenging, often leading to delayed recognition and treatment [3]. While most cases are sporadic, ALS arises from a complex interplay of genetic and environmental factors, including occupational exposures, lifestyle habits, and body mass index (BMI) [4]. Genetic mutations account for approximately 5–10% of cases, with C9orf72, SOD1, TARDBP, and FUS being the most commonly implicated genes [3]. These mutations disrupt various cellular processes, including RNA metabolism, protein homeostasis, mitochondrial function, and axonal transport, contributing to motor neuron degeneration [2]. Environmental exposures, such as heavy metals, pesticides, and occupational risks, have also been linked to ALS, although their exact contribution remains under investigation [5,6]. Lifestyle factors, including smoking and dietary habits, may further modulate disease risk and progression [7]. Systemic factors play a significant role in disease progression. Metabolic alterations, such as hypermetabolism and lipid dysregulation, exacerbate energy deficits, promote systemic inflammation, and accelerate neurodegeneration [8]. Similarly, gastrointestinal dysfunction and gut microbiota (GM) dysbiosis are increasingly recognized as contributors to ALS pathophysiology, driving immune dysregulation, compromising intestinal barrier integrity, and disrupting gut–brain axis signaling [9,10]. ALS progression is highly individualized and often complicated by malnutrition due to dysphagia, increased energy expenditure, and other disease-related factors, which further worsen prognosis [11,12]. Emerging evidence highlights the critical role of nutrition in ALS, both in prevention and therapeutic management. Tailored dietary interventions, along with strategies to support nutritional status, have shown potential to improve survival and quality of life. Furthermore, targeting gut microbiota (GM) through approaches such as probiotics, fecal microbiota transplantation (FMT), and dietary modifications may offer novel therapeutic opportunities. This review aims to summarize current knowledge on the role of nutrition in ALS, from prevention to disease management, while exploring microbiota-based strategies as promising avenues for improving outcomes.

## 2. Metabolic, Gastrointestinal and Microbiota Alterations in ALS

ALS is increasingly recognized as a multifaceted disease involving profound metabolic dysfunction, gastrointestinal (GI) disturbances, and GM dysbiosis, all of which interact with nutritional status and disease progression. Understanding these interconnected systems is essential in the context of a growing focus on nutrition as both a preventative and therapeutic strategy for ALS. This section highlights the critical metabolic, GI, and GM alterations in ALS to provide a base for exploring how nutrition and GM-targeted therapies could transform patient care.

### 2.1. Metabolic Dysfunction

Metabolic alterations are an ALS hallmark, significantly influencing disease progression and outcomes. Up to 50% of ALS patients exhibit a hypermetabolic state, marked by increased resting energy expenditure (REE), which contributes to weight loss and muscle wasting over time [13,14,15,16,17,18,19,20,21]. Notably, elevated REE often appears early in the disease, even before significant neuromuscular degeneration is evident [13,14]. Weight loss frequently begins up to a decade before motor symptoms, with rapid reductions correlating with poorer prognosis [12,22,23,24]. BMI is closely linked to ALS outcomes; lower BMI increases the risk of ALS developing, while higher BMI is associated with slower disease progression and longer survival [22,25,26]. A large prospective study of nearly 400,000 participants highlighted a significant association between metabolic syndrome (MetS) and ALS, especially in patients with BMI below 25 [27]. This relationship strengthens with the presence of additional metabolic abnormalities, implicating biomarkers such as alkaline phosphatase (ALP), cystatin C, and gamma-glutamyl transferase (γ-GT) [27]. In addition, gender differences influence metabolic factors; for instance, hypertension shows a stronger correlation with ALS risk in females [28,29]. Adipose tissue dynamics further underscore the metabolic complexity in ALS. Patients often experience reductions in overall fat mass, with contrasting changes in subcutaneous and visceral fat. Subcutaneous fat correlates positively with survival in males, while visceral fat, linked to insulin resistance and systemic inflammation, may exacerbate disease processes [30,31].

Energy expenditure is additionally impacted by shifts in white adipose tissue (WAT) and brown adipose tissue (BAT) activity. WAT “browning”, characterized by increased mitochondrial activity and uncoupling protein 1 (UCP1) expression, highlights altered lipid metabolism in ALS [32,33]. Dyslipidemia, often presenting as hyperlipidemia, is another metabolic feature of ALS. Elevated levels of fatty acids, sterol lipids, and triglycerides are detected in blood and cerebrospinal fluid (CSF), with distinct lipid signatures observed in both patients and animal models. These include increased cholesterol esters and decreased cardiolipin, mirroring shifts in energy use and mitochondrial dysfunction [34,35,36]. Elevated cholesterol and triglyceride levels have been associated with prolonged survival, underscoring their potential protective role [37,38]. A recent review by Ludolph et al. [39] provides further insights into these metabolic disturbances, emphasizing the complexity and clinical impact of hypermetabolism in ALS. They highlight that although hypermetabolism is consistently observed, its etiology remains largely unknown, necessitating a consensus approach for its identification. Authors also underscore the role of bioenergetic failure, driven by mitochondrial dysfunction, in the sensitivity of high-energy-demanding neurons to apoptosis. These findings align with evidence that dysregulated lipid and glucose metabolism, as well as systemic energy imbalances, are central to ALS progression. Our recent findings highlight a marked increase in medium-chain fatty acids (MCFAs) and a decrease in short-chain fatty acids (SCFAs) in ALS patients, with similar alterations observed in SOD1 mice. In this animal model, elevated MCFAs and decreased SCFAs correlate with more rapid disease progression, implicating these lipid shifts in the pathophysiology of faster-progressing ALS phenotypes [40,41]. It is well documented that mitochondrial dysfunction plays a central role in ALS-related metabolic dysregulation. Transactive response DNA binding protein 43 (TDP-43) mutations disrupt mitochondrial morphology and function, impairing fatty acid oxidation and exacerbating oxidative stress and inflammation [42,43]. Arachidonic acid, linked to disease progression, highlights the interplay between lipid metabolism and neurodegeneration [44]. Glucose metabolism is similarly impaired in ALS. Hypothalamic dysfunction disrupts energy balance, with structural defects and TDP-43 protein inclusions linked to weight loss and decreased BMI [45,46]. Fluoro-2-deoxy-d-glucose positron emission tomography (FDG-PET) (FDG-PET) imaging studies reveal hypometabolism in motor and frontal cortices alongside hypermetabolism in regions like the hippocampus, suggesting systemic glucose dysregulation [47]. Peripheral tissues, including muscle and adipose, exhibit shifts from glycolysis to fatty acid oxidation, contributing to energy deficits and systemic imbalance [48]. Moreover, organs such as the liver and pancreas show significant involvement in ALS-related metabolic abnormalities. Mitochondrial damage and inflammation in the liver, as well as β-cell loss in the pancreas, disrupt energy homeostasis and insulin regulation [49,50]. These systemic dysfunctions further exacerbate the hypermetabolic state, emphasizing the need for comprehensive research to clarify their roles in ALS progression. Overall, the metabolic dysregulation, encompassing lipid and glucose metabolism, mitochondrial dysfunction, and organ-specific alterations, highlights a complex interplay that significantly impacts ALS progression/outcome. Surely, further studies are critical to unravelling these mechanisms and exploring therapeutic strategies targeting metabolic pathways.

### 2.2. Gastrointestinal Symptoms and Gut Microbiota

GI and autonomic symptoms are significant but often underappreciated aspects of ALS, contributing to reduced food intake, quality of life, and so survival [51]. A spectrum of GI disturbances—including sialorrhea, constipation, acid regurgitation, dysphagia, and delayed colonic transit—affects a substantial amount of ALS patients, often correlating with disease severity and progression [52]. Sialorrhea, a debilitating condition affecting up to 50% of ALS patients, arises from impaired swallowing due to tongue spasticity, orofacial muscle dysfunction, and facial weakness rather than increased saliva production [53,54]. This condition significantly impacts quality of life, leading to complications such as aspiration pneumonia, a major cause of ALS mortality [55,56], and necessitates pharmacological or invasive management strategies [57].

Similarly, dysphagia affects approximately 25% of patients at disease onset and over 80% as ALS progresses, increasing risks of weight loss, aspiration pneumonia, and malnutrition [58]. Constipation, reported in up to 60.5% of patients, along with symptoms such as rectal tenesmus, hard stools, and borborygmus, further complicates disease management [52]. These symptoms often result from dysphagia-induced dietary changes, reduced fluid and fiber intake, physical inactivity, medication side effects, and psychological stress [59,60]. Autonomic nervous system involvement is also implicated, supported by pathological findings in the intermediolateral columns and Onuf’s nucleus, which suggest disruptions in the enteric nervous system (ENS) regulation of GI motor functions [60]. Experimental ALS models have demonstrated TDP-43 pathology in the gut, documenting ENS involvement even before motor symptom onset [61,62,63].

GM dysbiosis is a potential contributor to these GI disturbances, influencing luminal fluid balance, bile acid metabolism [64], short-chain fatty acid (SCFA) production [65], and intestinal motility. The vagus nerve, as a critical communication pathway between the gut and brain, mediates these interactions, with bacterial metabolites and neuropeptides affecting both enteric and central nervous systems [66,67]. Emerging evidence suggests that GI symptoms often precede motor symptoms, highlighting a potential link between GM dysbiosis and the disease’s underlying mechanisms [68]. The GM’s role in ALS pathogenesis has garnered increasing attention due to its connections with metabolic, immunological, and neurological alterations observed in patients. Dysbiosis has been implicated in ALS through multiple pathways, including immune modulation, oxidative stress, and neuroinflammation [9,69,70]. Specific bacterial taxa, such as Akkermansia muciniphila and Alistipes obesi, associated with lean phenotypes and weight loss, may contribute to metabolic changes commonly observed in ALS patients [71]. Additionally, pathogenic bacteria and their metabolites may enter systemic circulation via a compromised intestinal epithelial barrier (IEB), cross the blood–brain barrier (BBB), and exacerbate neurodegeneration [9,72]. These findings underscore the relevance of exploring the gut–brain axis and its influence on ALS pathology and progression. Animal model studies have provided valuable insights into the role of GM in ALS. For example, the superoxide dismutase (SOD1) G93A mouse model exhibited early microbiota shifts, leaky gut, and reduced butyrate-producing bacteria, all of which correlated with disease progression [10]. Remarkably, supplementation with Akkermansia muciniphila increased cerebrospinal nicotinamide levels, improved motor function, and extended survival in ALS mice [10]. Similarly, Burberry et al. demonstrated that microbiota modulation using antibiotics or fecal microbiota transplantation (FMT) significantly attenuated inflammation and extended survival in C9orf72^−/−^ ALS mice. These findings highlight the therapeutic potential of GM targeting to slow disease progression [73].

Parallel studies have similarly reported significant alterations in the GM of ALS patients, though results remain inconsistent due to small sample sizes, variable methodologies, and confounding factors such as diet and disease stage [59]. Notably, Mazzini et al. observed an increase in neurotoxic cyanobacteria in ALS patients [74], while many groups reported reductions in butyrate-producing bacteria and shifts in the Firmicutes/Bacteroidetes ratio [75,76,77,78]. A higher Firmicutes-to-Bacteroidetes ratio has also been linked to shorter survival [79], and Zhang et al., using Mendelian randomization, identified specific bacterial taxa such as Enterobacteriaceae and *Sutterella* as potential risk factors for ALS, implicating glutaminergic excitotoxicity in disease pathogenesis [80]. Despite these advancements, the mechanisms driving dysbiosis in ALS remain unclear. Dysbiosis may go before disease onset or arise as a secondary consequence. Factors such as diet, a critical determinant of GM composition, could contribute either before or after disease onset, especially in patients with bulbar dysfunction or reduced appetite. Alternatively, neurodegeneration itself may impair gut motility, or systemic immune alterations in ALS could exacerbate microbial imbalances [81]. Antibiotic exposure, another known disruptor of the GM balance, has also been associated with increased ALS risk and more severe phenotypes in both humans and animal models [82].

As previously reported, the consequences of GM dysbiosis in ALS are far-reaching, including immune dysregulation, increased toxin absorption, and metabolic abnormalities. These findings highlight the need for comprehensive, multidisciplinary ALS management and further research into the role of the GM and the gut–brain axis (GBA) in ALS pathology and progression. Interventions targeting gut dysbiosis—such as the use of probiotics, prebiotics, dietary modifications, and FMT—hold significant potential for restoring microbial balance and so improving patient outcomes. These approaches aim not only to enhance the abundance of neuroprotective and butyrate-producing bacteria, but also to mitigate systemic inflammation, optimize metabolic function, and strengthen the gut epithelial barrier. Emerging clinical trials and preclinical studies are beginning to evaluate the efficacy of these microbiota-based therapies, setting the stage for a deeper understanding of their role in ALS management.

## 3. Nutritional and Lifestyle Interventions in ALS Risk and Prevention

### Nutrition

Nutrition has emerged as a key modifiable risk factor with significant neuroprotective potential across various neurological disorders, including dementia, Parkinson’s and Alzheimer’s diseases [83]. Growing evidence underscores the role of specific dietary patterns, nutrients, and bioactive compounds in supporting neural health, mitigating oxidative stress, and reducing inflammation [83]. Recent research has extended this perspective to ALS, suggesting that specific dietary interventions and nutritional strategies may contribute to lowering the risk or delaying disease onset [84]. Different dietary regimens have been investigated for their potential use in reducing ALS risk. Adherence to the Mediterranean Diet (MD), characterized by the high consumption of fruits, vegetables, whole grains, nuts, seeds, olive oil, and fish, has been linked to a lower risk of neurodegenerative diseases, including ALS [85,86]. The diet’s rich content of antioxidants, such as vitamins E and C, omega-3 fatty acids, and anti-inflammatory compounds like polyphenols, supports neuronal health by reducing oxidative stress and inflammation [87,88]. Animal studies implementing extra virgin olive oil, a key MD component, have demonstrated its ability to reduce oxidative stress and nitric oxide production, potentially delaying ALS onset [89,90]. Clinical studies, such as those analyzing dietary patterns in ALS cohorts, suggest that higher adherence to the Mediterranean style correlates with reduced ALS incidence due to its ability to counteract neuroinflammatory processes and oxidative damage [85,91,92]. In addition to MD, it has been hypothesized that a plant-based diet could also play a role, but there is limited direct evidence connecting it to the ALS risk. Plant-based diets are generally rich in antioxidants, polyphenols, and anti-inflammatory compounds, which might help mitigate the oxidative stress and inflammation implicated in ALS pathogenesis [93,94]. In contrast, the consumption of a fiber-rich diet has not been consistently associated with a decreased ALS risk [93]. Vitamins have been shown to exhibit neuroprotective properties, like the COSMOS study on cognitive functions [39], mainly due to their antioxidant role, which is crucial for ALS prevention.

Research on the anti-inflammatory properties of vitamins C and D in neurodegenerative diseases has produced mixed results, with some studies showing little or no benefit. Although vitamins C and D are often praised for their neuroprotective qualities, some research shows how complicated and perhaps limited their effects may be. In models of Alzheimer’s and Parkinson’s, vitamin D has shown promise in reducing neuroinflammation and improving cognitive abilities; however, the precise mechanisms underlying these effects are still unknown [95,96]. According to some research, vitamin D may change pro-inflammatory microglial responses to anti-inflammatory responses, but its overall effect on neurodegeneration may not be substantial enough to halt disease progression [95]. Other studies show that vitamin C can improve motor function and reduce neuroinflammation in models of Parkinson’s disease, but the extent of the protective effect is still unclear [97]. Although vitamin C shows promise in reducing neuroinflammation, its efficacy may vary depending on the particular neurodegenerative context [97]. While vitamins C and D might play a role in inflammation and oxidative stress, their potential benefits in neuroprotection should be studied further, either alone or in combination with other treatments.

Regarding the antioxidant roles of other vitamins, tocopherol (vitamin E), a fat-soluble antioxidant, has demonstrated neuroprotective effects in preclinical studies, including its ability to prevent lipid peroxidation and reduce ferroptosis in motor neurons [94,98]. Clinical trials have linked an adequate intake of vitamin E, both through supplementation and diet, to lower ALS incidence [99,100]. Additionally, a study by Xia et al. suggests that vitamins D and E may diminish ALS risk by enhancing cellular protection mechanisms [101]. Anyway, vitamin E research has predominantly focused on other neurodegenerative diseases, leaving a void of systematic studies specifically targeting ALS [102], which has led to fewer observational studies and controlled trials focusing on the role of vitamin E in ALS. Despite these challenges, the potential role of vitamin E in the prevention of ALS remains an area of interest that deserves further investigation to elucidate its effects and mechanisms.

Regarding the possible downsides of vitamin E, research suggests that vitamins E and K may have counteracting effects on neurodegenerative diseases, mainly due to their functions in modulating oxidative stress. In several experimental settings, vitamin E, as a strong antioxidant, has been shown to preserve neurons and improve cognitive abilities. Studies are ongoing to elucidate potential interactions between vitamin K and neuroprotective pathways, especially in light of the benefits of vitamin E [103].

On its side, vitamin K, contained in green leafy vegetables largely present in the MD [104], is known for its role in blood clotting, but recent research reveals its impact on the central nervous system through extra-hepatic Gla proteins. Gas6, a vitamin K-dependent protein, plays a neuroprotective role by promoting cell survival, proliferation, and myelination in the central nervous system [105]. Vitamin K, particularly K2, activates Gas6 by facilitating the carboxylation of its glutamic acid residues [106]. Matrix Gla protein (MGP), another Gla protein, requires the activation of vitamin K, particularly K2, to prevent vascular and soft tissue calcification, which is critical for maintaining CNS vascular health [106]. Periostin is a Gla protein involved in bone formation and wound healing, but sources do not provide information on its direct effects on the CNS. Although the studies do not provide any evidence linking vitamin K and ALS, future research on vitamin K will attract more attention, given its role in improving cognitive functions. Among other nutrients, ergothioneine (ERGO) is a naturally occurring amino acid and a potent antioxidant found in some foods such as edible mushrooms [107]. Although mammals are unable to produce ERGO, they can absorb it through diet, and ERGO is particularly interesting because it accumulates in tissues remaining in the body for long periods. In addition, its potent antioxidant capacity allows it to scavenge free radicals, protecting neurons from oxidative damage [108]. Studies have shown that ERGO concentrations in blood components are lower in subjects with mild cognitive impairment (MCI), Alzheimer’s disease (AD), and Parkinson’s disease (PD). These characteristics, combined with its antioxidant and anti-inflammatory properties, make it a potential therapeutic agent for neurodegenerative disorders. However, more clinical trials are needed to fully understand its efficacy and safety in humans with neurodegenerative disorders. Further research is also needed to understand whether the reduction in ERGO levels is a cause or a consequence of the development of neurodegenerative disorders [109].

Regarding other dietary components investigated in relation to ALS risk, the relationship between magnesium intake and the risk of ALS was studied, but the findings do not support a protective effect [110]. Additionally, neither coffee nor tea consumption has been consistently associated with a reduced risk of ALS developing [111]. Furthermore, research suggests that diets high in fat, especially from animal sources, may elevate ALS risk by exacerbating oxidative stress and contributing to metabolic imbalances [112,113]. The elevated consumption of animal proteins and fats has been associated with increased levels of glutamate and LDL cholesterol, both of which are implicated in pathways that can promote neurotoxicity and inflammation [84]. Furthermore, other studies indicate that higher levels of cholesterol and its metabolites may raise the risk of ALS developing [114]. On the other hand, the consumption of polyunsaturated fatty acids (PUFA) and omega-3 rich food may help prevent or delay ALS onset [100,115].

Although human research presents some variability, these findings suggest that incorporating these dietary principles may help mitigate factors associated with ALS risk. While the diets discussed here suggest a promising link between reducing oxidative stress and inflammation in ALS and the increased intake of antioxidant and anti-inflammatory rich foods, more research is needed to directly compare their impacts on mechanisms underlying ALS onset.

## 4. Nutritional Management in ALS: Improve Quality of Life and Slowing Disease Progression

Ensuring and maintaining an adequate nutritional status from the initial stages of the disease is essential to increase ALS patients’ quality of life, prolong their survival and prevent the occurrence of medical conditions related to malnutrition, mainly protein deficiency [116]. Most ALS patients experience some degree of malnutrition as the disease progresses, with 16% to 55% showing signs of malnutrition at the time of diagnosis [110]. Of note, malnutrition at diagnosis and hypermetabolism are associated with less survival [12,21,117]. According to ESPEN guidelines, nutritional deterioration in ALS patients is caused by several factors related to the disease’s pathophysiology [118]: (i) bulbar neuron degeneration leads to difficulties with chewing, oral preparation, meal completion time, and dysphagia; (ii) anorexia, usually attributed to psychosocial distress, depression, and multiple treatments; (iii) weakness in the abdominal and pelvic muscles, reduced physical activity, self-restriction of fluids, and a low-fiber diet can cause constipation, which indirectly reduces food intake; (iv) ALS patients may have increased energy requirements despite decreased lean body mass due to factors like increased breathing effort, lung infections, and other not well-established factors; (v) cognitive dysfunction, primarily frontotemporal dementia.

Nutritional status can influence the disease prognosis. As previously discussed, anthropometric measures like BMI and weight loss are related to disease progression and survival, with a low BMI or significant weight loss at diagnosis being associated with a worse prognosis [11,12]. A recent prospective study examined the prevalence and prognostic value of weight at the time of ALS diagnosis: the study involved 2420 patients, 67.5% of whom reported losing weight at first referral, and it found that weight loss was a predictor of survival, with a 23% increased risk of death for every 10% increase in weight loss relative to body weight [119]. Comparable outcomes were observed in 63 ALS patients, where a subgroup of patients with weight loss greater than 10% had a mean disease duration that was considerably shorter than that of the other individuals [120]. Furthermore, patients who lost less than 5% of their normal body weight in the six months before receiving an ALS diagnosis had a longer median survival than those who lost more weight, suggesting that stable weight at diagnosis is crucial [12]. Body composition analysis using bioelectrical impedance analysis (BIA) has shown that decreases in both fat-free mass (FFM) and fat mass (FM) predict poor outcomes [121,122]. A study on 53 ALS patients found that a higher average monthly change in body fat percentage and continued oral food intake are significant prognostic factors for longer survival [123]. The average caloric intake of ALS patients is estimated to be 15% below recommended levels [124]. Inadequate support needed during meals, dysphagia, and taste abnormalities can all contribute to inadequate food intake [24]. Consequently, a crucial component of ALS patient care is providing suitable nutritional care and doing appropriate nutritional assessments [125,126]. Ludolph et al. also stress the therapeutic potential of high-calorie diets and personalized nutritional interventions, with ongoing clinical trials aiming to optimize energy balance in ALS patients [39].

### 4.1. Comprehensive Nutritional Assessment in ALS

A thorough nutritional assessment is advised for ALS patients at the time of diagnosis and at follow up, every three months [118]. Table 1 provides a comprehensive overview of the key aspects of nutritional assessment essential to identifying malnutrition for ALS patients.

Even though various tools have been presented for the diagnosis, there is currently no gold standard for ALS patients. Among these, we find the Global Leadership Initiative on Malnutrition (GLIM) criteria and the Subjective Global Assessment (SGA). Lòpez-Gòmez et al. assessed 93 ALS patients for malnutrition; using the SGA, 29% of patients were classified as well-nourished (grade A), 46.3% as moderately malnourished (grade B), and 24.7% as severely malnourished (grade C). According to the GLIM criteria, 48.4% of patients were identified as malnourished [116]. The study found that patients with poorer nutritional status had a lower median survival time, with significant differences observed using both the SGA and GLIM criteria. For instance, patients in SGA grades B and C had a median survival of 12 months compared to 20.5 months for those in grade A. Similarly, severe malnutrition according to GLIM criteria was associated with a median survival of 18 months versus 20 months for those with no severe malnutrition. The authors concluded that ALS patients often experience severe nutritional deterioration, and better nutritional status is associated with longer survival times. This underscores the importance of an early and accurate assessment of nutritional status using tools like SGA and GLIM criteria to improve patient outcomes [109]. However, applying the GLIM criteria to ALS patients requires understanding the biases due to the disease’s characteristics, especially in the first stages [127]. Initial weight loss may indicate malnutrition, but could also mirror factors like hypermetabolism and rapid muscle atrophy, which contribute to speeding up disease progression [127].

Assessing the role of neuroinflammation, estimating total energy requirements and identifying nutritional intake remains a challenge in the GLIM framework applied to ALS [127]. Sixty-seven percent of the dietitians surveyed in a UK-wide study evaluating dietary practices reported using a malnutrition screening tool, with the Malnutrition Universal Screening Tool (MUST) being the most often mentioned [128]. Although anthropometric parameters, such as BMI and weight history, are crucial to evaluating nutritional status, they do not provide information on the body compartments, and thus do not allow for the evaluation of potential losses in FFM or increases in FM. Both at the time of diagnosis and at follow-up, the assessment of body composition is critical [118].

In clinical practice, frequently used tools include body circumferences and skinfold thickness measurements [129,135]. Using the Durnin and Womersley procedures, it is also feasible to estimate body density, FFM and FM from the measurements of four cutaneous skinfold thicknesses (tricipital, bicipital, suprailiac and subscapular) [135,136]. Conventional methods for measuring body composition, such as DXA [137,138] and BIA [139,140], have limitations including high costs, limited availability, and assumptions regarding hydration status [141]. DXA is the gold standard for defining body composition, although it is expensive, usually unavailable, and hardly utilized in ALS management [142]. When compared to DXA, BIA with a validated formula offers a straightforward, quick, and accessible way to evaluate the body composition of ALS patients in the context of therapeutic practice [143]. Additionally, these methods often require the patient to lie supine, which can be difficult for ALS patients experiencing orthopnea due to bulbar or respiratory issues [141]. Therefore, a simple, practical, and cost-effective method for estimating body composition for the nutritional assessment of ALS patients is needed. Tandan et al. developed an equation incorporating sex, age, BMI, and bulbar-onset status to estimate body fat, which was validated against DXA in 104 patients, showing minimal variance [144]. The findings suggest that the new equation provides a reliable method for estimating body composition, allowing for targeted nutritional interventions to improve energy intake and potentially enhance patient outcomes in ALS. The study also showed that, in a larger cohort of 314 patients, low baseline FM and the loss of FM and FFM over six months were significantly associated with decreased survival [144]. Phase angle (PA), another physical measure derived from BIA, has been suggested as a survival-predictive factor for ALS patients and as a malnutrition index [145]. In a study of 168 ALS patients, PA was significantly lower in ALS patients compared to healthy controls, and even lower in malnourished ALS patients [145]. Furthermore, a loss of one degree of PA was associated with a 29% higher chance of mortality [12]. During the nutritional assessment, it is essential to evaluate serum parameters such as albumin, creatinine and lipid profile [118]. Serum albumin and creatinine are separate indicators of prognosis in both genders with ALS; albumin is associated with an inflammatory state, while creatinine indicates the waste products of muscle [131]. A decrease in serum albumin was associated with a higher risk of death [131]. ALS patients frequently exhibit increased serum lipid and cholesterol levels [38,146]. Hyperlipidemia was reported to be neuroprotective and related to a significantly better outcome in ALS [38,147]. Nevertheless, other studies document that cholesterol and its metabolites may mediate oxidative stress in motor neurons [148]. Moreover, nutritional assessment must necessarily be supplemented with the clinical evaluation of factors such as dysphagia, chewing difficulty and hypermetabolism, which may contribute to compromising the nutritional status, both at the time of diagnosis and at follow-up, usually every 3 months [118].

The hypermetabolism assessment of increased REE, which often contributes to worsening the malnutrition picture, is complex [21]; clinics typically do not have access to indirect calorimetry, and so REE is estimated using equations that are less accurate and do not take into account the individuality of each patient [132]. The Ireton–Jones equations consistently overestimated REE, while the Harris–Benedict and Mifflin–St Jeor equations produced clinically appropriate estimations of REE across the illness spectrum [133].

Furthermore, ALS patients frequently experience anorexia due to a combination of psychosocial factors, depression, and medical treatments [118,149]. These issues, along with difficulties in swallowing, chewing and taste disorders, significantly impairs their dietary intake [141,142]. For this reason, a relevant aspect of nutritional assessment is the collection and analysis of eating habits. In the studies investigating the nutritional adequacy of ALS patients, the most frequently used method is the 24-h recall, a survey wherein the patient or their caregiver is asked to describe a day’s nutrition, usually from the previous day [130,134]. Given the growing evidence of the GM’s involvement in ALS onset and progression, including its assessment into the clinical and nutritional evaluation of patients may prove beneficial. The early identification of alterations in GM composition and function could represent an opportunity for future interventions aimed at modulating these changes, potentially improving disease progression and alleviating associated symptoms [9,70].

### 4.2. Nutritional Interventions and Treatment Strategies

Based on nutritional and clinical assessment, the intervention aims to achieve or maintain optimal nutritional status and address disease-related complications (such as dysphagia, hypermetabolism, chewing difficulties and gastrointestinal symptoms) [150]. Adequate nutritional support (for maintaining or gaining weight) can improve disease outcomes [150]. Weight gain is recommended for patients with a BMI less than 25 kg/m^2^, stabilization of weight for those with a BMI between 25 and 35 kg/m^2^, and weight loss for patients with a BMI over 35 kg/m^2^ to facilitate active and passive mobilization [118]. Weight management must consider the metabolic variations associated with the patient’s clinical condition [151]. In the absence of indirect calorimetry, energy requirements in ALS patients should be approximated. Estimates for calculations should be made using roughly 30 kcal/kg of body weight, considering changes in physical activity and weight and body composition [132,151]. Non-invasive ventilation typically results in a lower REE compared to spontaneous breathing or the values predicted by the Harris–Benedict equation [152,153]. If indirect calorimetry is missing, energy requirements should be estimated at 25–30 kcal/kg of body weight or calculated using the Harris–Benedict equation, and then adjusted based on changes in body weight and the clinical condition [152,153]. ALS patients who experience fatigue and prolonged mealtimes should receive dietetic counseling on enriching their meals with high-calorie, low-volume food [118,154]. Additionally, the consistency of the food should be adjusted to match their chewing and swallowing abilities [155]. If they cannot meet their nutritional needs through oral intake alone, nutritional treatment may include oral nutritional supplements or artificial nutrition [155,156]. While not yet confirmed in the literature, enteral nutrition (EN) seems to enhance the quality of life for patients with ALS [157]. EN is preferable to parenteral nutrition (PN) due to its lower risk of infection, easier management, and ability to maintain gastrointestinal trophism and function.

In acute situations, PN can be used if EN is contraindicated or not feasible [156]. EN, usually with gastrostomy, should be considered early and reviewed regularly as ALS progresses, based on the evolution of swallowing difficulties, safety, and efficacy [158,159]. Decisions regarding gastrostomy placement should be suggested by the detection of dysphagia, prolonged mealtimes, weight loss, poor respiratory function, risk of choking, and the patient’s preferences. It is advisable to perform the gastrostomy before significant weight loss and the severe impairment of respiratory function occur [38,159]. In addition to its therapeutic impact, the dietary approach may influence ALS progression by affecting underlying mechanisms such as oxidative stress, mitochondrial dysfunction, inflammation, and GM composition and function.

As previously reported, in addition to its preventative role, the MD has been proposed as a potential therapeutic approach for managing neurodegenerative disease due to its capacity to mitigate oxidative stress associated with neuronal death [86]. De Paola et al. demonstrated that extra virgin olive oil reduced neurodegeneration in ALS mouse models by attenuating nitric oxide production from activated glia and downregulating the TLR4 signaling pathway [90]. Furthermore, Oliván et al. found that an 8-week supplementation with extra virgin olive oil improved ALS pathology and delayed its onset in mice compared to a standard chow diet [89]. Carrera-Julia et al. reported that ALS patients following a 4-months MD enriched with antioxidants such as nicotinamide riboside (NR) and pterostilbene (PTER), or a ketogenic MD incorporating coconut oil, showed significant improvements in anthropometric measures compared to control groups [160]. This evidence suggests that diets rich in antioxidants may experience decreased inflammation and potentially improved ALS symptoms. ALS patients often consume inadequate daily caloric quantities, which underscores the potential need for high-calorie diets to stabilize body weight [124]. Furthermore, high-fat diets have also been suggested as a strategy to slow disease progression in the mutant superoxide dismutase 1 (SOD1) mouse model [161]. In this study, the intervention began at 75 days of age and continued for the mouse’s lifespan. The mechanism by which increased dietary fat, began at 60 days of age and continued for the mouse’s lifespan, prolongs survival in the SOD 1 mouse model remains unclear. Fergani et al. found that a diet with 21% butter fat normalized serum cholesterol levels, which were reduced in these mice when fed a regular diet [162]. Phospholipids and cholesterol are crucial for axonal membrane assembly, and cholesterol biosynthesis decreases in the peripheral nerves during degeneration and regeneration [163]. In peripheral nerve injury models, there is a significant increase in low-density lipoprotein (LDL) receptor expression, enabling the regeneration of nerves to import cholesterol for axonal repair [163]. Thus, elevated dietary fats may increase circulating LDL levels, potentially enhancing the survival of peripheral motor neurons. Moreover, epidemiological data from humans suggest that higher cholesterol levels might be linked to improved ALS survival, although this correlation was not observed when overall nutritional status, as documented by body mass index, was considered [24,38]. Zhao et al. investigated a ketogenic diet (comprising 60% fat, 20% carbohydrate, and 20% protein) in the SOD1 mouse model. The treatment began at 60 days of age and continued for the mouse’s lifespan. Although the study did not show a significant increase in survival, they observed improved functionality [164]. Additionally, they demonstrated increased ATP production from mitochondria purified from an ALS mouse spinal cord when treated with β-hydroxybutyrate [164]. The same research group also reported that caprylic acid treatment (a medium-chain triglyceride metabolized into ketone bodies), started at 10 weeks of age, appeared to enhance mitochondrial function and increase motor neuron numbers in the ALS mouse model, although it did not result in an overall survival benefit [165]. Ketogenic diets, characterized by high fat, moderate protein, and very low carbohydrate intake, have been identified as an effective therapeutic intervention for several neurodegenerative diseases, including medication-resistant epilepsy, Alzheimer’s disease, and Parkinson’s disease [166].

Research utilizing human-induced pluripotent stem cells and experimental familial ALS models has established a strong connection between mitochondrial dysfunction and the oxidative stress and DNA damage, which are underlying mechanisms for the neurodegeneration observed in ALS [167]. Ketone metabolism has been shown to increase the production of essential citric acid cycle substrates, such as acetyl coenzyme A, and to diminish mitochondrial free radical generation [168]. Consequently, ketogenic diets may offer neuroprotection and potentially slow the progression of motor neuron damage in ALS. However, contrasting results have been observed in a controlled study, suggesting that a high-calorie diet with increased carbohydrate intake was associated with greater survival and more positive outcomes compared to a high-fat diet [169]. Thus, while different dietary approaches might offer short-term benefits in managing body weight and potentially influencing disease progression, the overall effects and implications remain controversial and warrant further investigation.

## 5. Therapeutic Potential of Gut Microbiota Modulation

Research has increasingly shown that the gut microbiota play a crucial role in the pathogenesis of neurodegenerative disease, including ALS, by directly or indirectly influencing the gut–brain axis [170]. Alterations in the GM composition can lead to neuroinflammation, oxidative stress, and disruptions in the intestinal barrier, all of which contribute to ALS development and progression. These changes impair normal neuron function and exacerbate the neurodegenerative processes characteristic of ALS [170]. Given the evidence supporting the GM’s role in ALS, therapeutic strategies targeting GM modulation, such as probiotics, dietary interventions, and even fecal microbiota transplantation, are being explored as potential treatments to improve the GBA, mitigate disease progression and improve patients’ quality of life.

### 5.1. Dietetic Treatment

Disease progression can be slowed down by modifying the GM through nutrition and by changing the GBA’s signaling pathway from the bottom up [170]. Currently, the role of the diet in modulating the GM in the ALS condition still needs to be further investigated. To date, supplementation with bioactive compounds, particularly antioxidants, has been proposed to decrease the progression of neurodegenerative disease [171]. Among the antioxidants that have been successfully utilized in ALS, a treatment of EGCG (epigallocatechin gallate), beginning at 60 days of mouse’s life, stands out for its neuroprotective effects on motor neurons [172]. This protection is associated with the regulation of glutamate levels, which helps prevent the misfolding of the SOD1 protein [173]. However, its effects on the microbiota have not yet been studied. Curcumin has also yielded positive outcomes for ALS patients by reducing oxidative stress through various molecular mechanisms [174]. It improves the GM composition, notably affecting bacteria that are typically altered in ALS, such as Escherichia, Enterobacter, Clostridium, and Bacteroides [76,175,176]. In particular, a study investigated the effects of turmeric and curcumin supplementation (1 g daily for 8 weeks) on gut microbiota, finding significant alterations in microbiota composition in both curcumin and turmeric groups compared to placebo [176]. Yip et al. found that eicosapentaenoic acid (EPA), an omega-3 polyunsaturated fatty acid, did not provide therapeutic benefits in ALS [177]. Although EPA treatment reduced microglial cell activation, it decreased the lifespan of the mice and did not prevent motor neuron loss. Furthermore, EPA treatment, initiated at 60 days of mouse life, resulted in increased levels of neurotoxic byproducts, such as microglial 4-hydroxy-2-hexenal, in the spinal cords of the mice [177]. However, a greater number of studies specifically analyzing the effect of antioxidant supplementation or other bioactive compounds on the GM in ALS are needed.

### 5.2. Prebiotics

Prebiotics are non-digestible food components, mainly dietary fibers, that promote the growth and activity of beneficial microorganisms in the gut, thereby enhancing overall gut health and well-being [178]. Song et al. reported that administering galacto-oligosaccharides (GOS)-rich yogurt to 8-week-old SOD1-G93A transgenic mice significantly influenced the ALS progression and extended the animals’ lifespan [179]. The GOS treatment not only improved mitochondrial function in skeletal muscles but also reduced muscle denervation, atrophy and neuroinflammation [179]. Promising results were found in other neurodegenerative diseases, such as Alzheimer’s Disease (AD). Zhang et al. demonstrated that a 6-week oral administration of the GOS not only increased the relative abundance of *Lactobacillus*, but also mitigated neuroinflammation and cognitive impairment in transgenic AD mice [180]. Similarly, the use of fructo-oligosaccharides (FOS), whether alone or in combination with GOS, enhanced the growth of *Bifidobacterium* and ameliorated the AD pathology [180]. These beneficial effects were partially attributed to the downregulation of signaling pathways that are common between the colons and cortices of the mice, indicating that the modulation of the GBA could significantly impact CNS pathology [180]. However, the current preclinical work on the effectiveness of prebiotics in ALS is still in its early stages. More research is necessary to definitively establish whether prebiotic compounds are effective for ALS therapy.

### 5.3. Probiotics

Probiotics are live microorganisms that confer a health benefit to the host, through their influence on GM composition and function [181]. This modulation is mandatory for maintaining the integrity of the intestinal epithelial barrier, inhibiting pathogenic organisms, and regulating immune responses [181]. Empirical evidence shows that the GM composition and metabolites exert a profound impact on immune system functionality [182]. Additionally, there is a recognized interplay between immune responses and central nervous system function [182]. SCFAs, which are critical metabolites produced by the GM, are instrumental in modulating immune responses and serve as key signaling molecules within the GBA [182]. Probiotic administration has been shown to increase the abundance of SCFA-producing microorganisms in the gut [183]. For these reasons, probiotics have been proposed as a therapeutic strategy for ALS. Building on this premise, a prospective longitudinal study by Di Gioia et al. investigated the effects of a 6-month probiotic treatment in ALS patients. The study confirmed that the GM of ALS patients differs significantly from that of healthy controls, with a progressive decline in microbial diversity as the disease advances. Although probiotic administration altered the GM’s composition, it failed to restore diversity to levels seen in healthy controls or influence disease progression, as measured by the ALS Functional Rating Scale-Revised (ALSFRS-R) [74].

Chen et al. highlighted that ALS patients show elevated interferon-gamma (IFN-γ) levels from NK and CD8+ T cells, contributing to immune-related damage in the central nervous system and peripheral organs [184]. To counteract this, researchers developed a probiotic treatment strategy to increase anti-inflammatory interleukin-10 (IL-10) using specific probiotic strains. The results show that the probiotic strain Al-Pro (AJ3) had the lowest IFN-γ/IL-10 ratio and IFN-γ secretion compared to other formulations, indicating its potential effectiveness in reducing autoimmunity and protecting motor neurons in ALS by modulating IFN-γ levels and immune cell activity [184].

Blacher et al. found that restoring mitochondrial function through the administration of *Akkermansia muciniphila* was crucial for alleviating motor symptoms in ALS SOD1-G93A mice [10]. A recent study explored the impacts of sixteen probiotic formulations of *Caenorhabditis elegans* strains expressing human ALS-related proteins FUS and TDP-43 [185]. While most probiotics showed minimal effects, *Lacticaseibacillus rhamnosus* HA-114 significantly delayed neurodegeneration and prevented paralysis in these ALS models, but not in wild-type strains, suggesting its specific relevance to ALS pathology. Further research revealed that HA-114 countered ALS-related metabolic dysfunction, including impaired β-oxidation and disrupted energy homeostasis, through its unique fatty acid content, which restored energy metabolism independently of the carnitine shuttle [185].

### 5.4. Postbiotics

Postbiotics are bioactive compounds produced by GM, which include metabolic byproducts (e.g., SCFA), cell-wall fragments, functional proteins and extracellular polysaccharides [186]. These compounds can confer health benefits to the host by modulating the GM, enhancing the immune response, and exerting anti-inflammatory effects [186]. A 13-week administration of 2% sodium butyrate reduced microglia in the spinal cords of SOD1-G93A mice, and was associated with lower circulating levels of proinflammatory IL-7 and LPS, as well as inducing alterations in microbial carbohydrate and amino acid metabolism [187]. Furthermore, Zhang et al. evaluated butyrate as a potential ALS therapy [188]. Butyrate treatment started at age 63 days, and finished after 2.5 months. Using G93A transgenic mice, the research found that butyrate restored intestinal microbial balance, improved gut integrity, delayed ALS progression, and extended lifespan compared to untreated controls. Butyrate also reduced abnormal Paneth cells and decreased the aggregation of the G93A SOD1 protein, suggesting it could be a promising treatment for ALS by addressing intestinal dysbiosis [188]. Neuroprotective effects were further attributed to sodium phenylbutyrate, which modulates the expressions of several anti-apoptotic genes [113]. Besides inhibiting histone deacetylase, phenylbutyrate administration significantly upregulated nuclear factor-kappaB (NF-kB) and beta-cell lymphoma 2 (Bcl-2) expression, thereby blocking caspase activation and subsequent motor neuron death [189]. Although phenylbutyrate treatment alone significantly delayed disease progression, Signore et al. found that its combination with riluzole, a widely recognized drug for ALS treatment, was most effective in prolonging survival in G93A transgenic ALS mice [190]. Moreover, this combination mitigated body weight loss and preserved grip strength [190]. Despite these encouraging results, larger-scale studies are needed to determine the long-term efficacy of combined therapies and their potential applicability to ALS patients. Furthermore, in studies on postbiotic and probiotic supplementation, a significant limitation is the lack of a clearly defined treatment duration. This inconsistency makes it challenging to evaluate the long-term effects and overall efficacy of these interventions.

### 5.5. Fecal Microbiota Transplantation

Fecal microbiota transplantation involves transferring healthy gut bacteria from the feces of a donor into the gastrointestinal tract of a patient [191]. This procedure aims to reestablish a healthy microbial community in the patient’s gut, with the intention of treating both intestinal and non-intestinal conditions [191]. Yan et al. reported the use of FMT in two late-onset ALS patients who were severely affected and required tracheostomy and mechanical ventilation [192]. Following two rounds of FMT, both patients showed significant improvements in respiratory function, allowing them to be weaned off mechanical ventilation. Additionally, their muscle strength, swallowing ability, and reduction in muscle fasciculations improved. Metagenomic and metabolomic analyses revealed an increase in beneficial gut bacteria and changes in metabolite levels, suggesting the potential therapeutic benefits of FMT for ALS and offering new insights into the disease [192]. Lu et al. reported on an ALS woman who benefited from a washed microbiome transplant (WMT) administered via a trans endoscopic bowel tube [193]. Although her condition initially improved, she later experienced a scalp injury treated with antibiotics, which worsened her ALS. However, a subsequent WMT effectively reversed this decline and halted the disease’s progression [193]. A new clinical trial is currently underway to assess the impact of FMT on GBA and immune function in ALS patients [194,195].

## 6. Conclusions and Future Perspectives

Emerging evidence highlights the critical interplay between metabolic alterations and gut microbiota dysbiosis in the ALS pathogenesis. Metabolic dysfunction, including hypermetabolism, lipid imbalances, and glucose metabolism disruptions, exacerbates the disease’s progression, while GM dysbiosis contributes to systemic inflammation, oxidative stress, and neurodegeneration through the GBA. These findings underscore the interconnected roles of systemic metabolic health and microbiota composition in ALS pathology.

Nutritional interventions represent a promising avenue for both the prevention and therapeutic management of this disease. Dietary patterns rich in antioxidants, omega-3 fatty acids, and anti-inflammatory compounds, such as the MD, have shown potential in mitigating oxidative stress and inflammation, improving metabolic balance, and enhancing patient outcomes. Furthermore, GM-targeting therapeutic strategies—such as probiotics, prebiotics, postbiotics, and FMT—offer novel opportunities to restore microbial balance, reduce neuroinflammation, and optimize GBA signaling.

We acknowledge, however, that the current literature lacks a comprehensive integration of evidence directly linking specific nutritional interventions to microbiota changes in ALS. While studies demonstrate the effects of diet and supplementation on ALS progression and the potential of microbiota modulation as a therapeutic strategy, few investigations simultaneously address both aspects. This limitation underscores the need for future research that combines these domains to establish causal relationships and elucidate underlying mechanisms.

Future research should focus on the following: (i) identifying specific dietary regimens and supplements tailored to ALS patients’ metabolic and microbiota profiles to prevent or mitigate disease progression; (ii) exploring personalized microbiota-based therapies to improve neuroinflammation and GBA interactions, while understanding the causal relationships between microbiota dysbiosis and ALS onset; (iii) integrating immunonutrition with microbiota-targeted strategies, such as combining dietary interventions with probiotics or postbiotics, to amplify therapeutic benefits; (iv) conducting large-scale, longitudinal studies to evaluate the efficacy and safety of these interventions, as well as their long-term impacts on quality of life and survival. The integration of nutrition and microbiota modulation into ALS management could transform care paradigms for neurodegenerative and neuroinflammatory diseases. By addressing the gaps in understanding the interplay between nutritional supplementation and microbiota modulation, future studies hold the potential to uncover innovative and effective strategies to slow disease progression and improve patient outcomes.

## Figures and Tables

**Table 1:** Nutritional assessment of ALS patients: methods, limitations, and clinical implications. Key aspects of nutritional assessment in ALS patients, highlighting the tools and methods used, their limitations, and their relevance in clinical practice for improving patient outcomes.

Assessment	Tool/Method	Clinical Implication	Limitations
Malnutrition Screening	GLIM criteria (phenotypic/etiologic combination), SGA (grades A, B, C) [116]	Useful for early targeted nutritional interventions [116]	Biases due to the disease’s characteristics [127]. No gold standard or specific tool for ALS [118]
Anthropometric Assessment	BMI, body weight, weight loss, body circumferences (BC), skinfold thickness (ST) [118,128,129]	Evaluation and monitoring; prevention of worsening of nutritional status and prognosis [118]	Operator-dependent variability (BC, ST). Not accurate indicators of overall nutritional status [118]
Body Composition Analysis	DXA, BIA (with validated formula), skinfold thickness (Durnin and Womersley) [118]	Estimation of FFM/FM. Evaluation of prognosis and survival (PA) [112,130]	Cost (DXA), availability (DXA), difficulty of supine position in patients with orthopnea, abnormal fat distribution (ST), unilateral effects of ALS (ST) [131,132]
Serological Parameters	Albumin, creatinine, lipid profile [125]	Prognosis indicators [131]	Not specific to nutritional status [131]
Swallowing and Chewing Ability	Structured questionnaires, water swallow test, volume–viscosity swallow test, videofluoroscopy, flexible endoscopic evaluation [118]	Early identification of dysphagia and chewing difficulties to adjust texture and nutritional intake. Prevention of respiratory infections [118]	Lack of ALS-specific standards [118]
REE/Hypermetabolism	Indirect calorimetry, predictive equations (Harris–Benedict, Mifflin–St Jeor) [118]	Estimating energy needs for personalized nutritional intervention [21,118]	Poorly accurate equations. Limited access to calorimetry [21,132,133]
Dietary Habits	24-h recall [130,134]	Assessment of nutritional adequacy, identification of dietary deficiencies [130,134]	Risk of inaccuracies in collected data
Microbiota Alteration	Analysis of microbiota composition and functionality [9,70]	Potential for targeted interventions to modulate the microbiota and improve disease progression.	Methods not yet integrated into clinical practice

GLIM, Global Leadership Initiative on Malnutrition; SGA, Subjective Global Assessment; BMI, Body Mass Index; DXA, Dual-energy X-Ray Absorptiometry; BIA, Bioelectrical Impedance Analysis; FFM, Fat-Free Mass; FM, Fat Mass; PA, Phase Angle.

## Data Availability

No new data were created or analyzed in this study. Data sharing is not applicable to this article.
